# Respondent driven sampling of wheelchair users: A lack of traction?

**DOI:** 10.12688/f1000research.8605.2

**Published:** 2016-08-26

**Authors:** John A. Bourke, Philip J. Schluter, E. Jean C. Hay-Smith, Deborah L. Snell

**Affiliations:** 1 School of Health Sciences, University of Canterbury, Canterbury, 8140, New Zealand; 2 Burwood Academy of Independent Living, Christchurch, 8140, New Zealand; 3 School of Nursing, Midwifery and Social Work, The University of Queensland, Brisbane, 4072, Australia; 4 Rehabilitation Teaching and Research Unit, University of Otago Wellington, Wellington, 6242, New Zealand; 5 Department of Orthopaedic Surgery and Musculoskeletal Medicine, University of Otago Christchurch, Christchurch, 8140, New Zealand

**Keywords:** Wheelchair users, Disability, Respondent driven sampling, Social epidemiology, Sampling approaches

## Abstract

*Background*: Internationally, wheelchair users are an emerging demographic phenomenon, due to their increased prevalence and rapidly increasing life-span. While having significant healthcare implications, basic robust epidemiological information about wheelchair users is often lacking due, in part, to this population’s ‘hidden’ nature. Increasingly popular in epidemiological research, Respondent Driven Sampling (RDS) provides a mechanism for generating unbiased population-based estimates for hard-to-reach populations, overcoming biases inherent within other sampling methods. This paper reports the first published study to employ RDS amongst wheelchair users.

*Methods*: Between October 2015 and January 2016, a short, successfully piloted, internet-based national survey was initiated. Twenty seeds from diverse organisations were invited to complete the survey then circulate it to peers within their networks following a well-defined protocol. A predetermined reminder protocol was triggered when seeds or their peers failed to respond. All participants were entered into a draw for an iPad.

*Results*: Overall, 19 people participated (nine women); 12 initial seeds, followed by seven second-wave participants arising from four seeds **.** Completion time for the survey ranged between 7 and 36 minutes. Despite repeated reminders, no further people were recruited.

*Discussion*: While New Zealand wheelchair user numbers are unknown, an estimated 14% of people have physical impairments that limited mobility. The 19 respondents generated from adopting the RDS methodology here thus represents a negligible fraction of wheelchair users in New Zealand, and an insufficient number to ensure equilibrium required for unbiased analyses. While successful in other hard-to-reach populations, applying RDS methodology to wheelchair users requires further consideration. Formative research exploring areas of network characteristics, acceptability of RDS, appropriate incentive options, and seed selection amongst wheelchair users is needed.

## Introduction

Robust epidemiological research generally requires data collection from representative samples of the population of interest, and effective modes of sampling contact are essential ^1^. Such effective modes can be difficult in hard-to-reach populations where no (or inadequate) sampling frames exist. Traditional chain-referral sampling approaches are inherently biased in their participant selection methods; a bias that is compounded as recruitment waves continue. An appealing alternative, Respondent Driven Sampling (RDS), was developed to counter these biases by employing specific data collection and statistical analysis methods which enable the derivation of valid population-based estimates ^2– 4^. RDS has traditionally been used to sample from ‘hidden’ populations with inadequate sampling frames, such as those with greater risk of HIV, including injecting drug users ^5, 6^.

In brief, RDS is initiated by recruiting a handful of individuals who serve as ‘seeds’. After completing the survey, seeds are then invited to recruit their peers to complete the same survey. To enhance recruitment, RDS employs ‘dual incentives’ whereby individuals are rewarded (usually monetary ^7^) for both their participation, and the participation they can elicit from the person(s) they recruit ^2^. For RDS to provide unbiased population estimates, several assumptions need to be satisfied. Unlike convenience sampling, RDS requires that recruitment chains are traceable (via recruitment codes), that participants can provide an estimate of their network size (the number of people a person knows in the target population), and that participant’s recruit randomly from their networks ^4^. If these RDS assumptions are met, at some point sampling will reach a state of equilibrium, and useable unbiased data – independent from the initial seeds – can be gleaned.

Equilibrium is deemed to have been reached when there is relatively little variation in the sample proportions of key participant characteristics (such as age or sex) between successive measurement waves. The threshold for variation tolerance is determined prior to RDS implementation and a value of 2% is commonly employed ^8, 9^. Equations for calculating equilibrium are described by Heckathorn (2002) ^10^. Data produced before sampling equilibrium is reached are termed ‘out-of-equilibrium’ and are normally discarded due to their inherent biases. Unbiased population-based estimates use ‘in-equilibrium data’, data generated after equilibrium is reached so that recruitment theoretically represents a probabilistically determinable sample of network members.

Due to its appeal, the use of RDS has rapidly increased in two decades, with over 120 RDS studies reported in more than 20 countries with over 30,000 participants ^7^. Despite wider adoption of RDS, and its successful application in many topic areas, concerns have been raised regarding whether RDS estimates hold in practice. For instance, some RDS estimates are more variable than expected ^11^, and some sampling patterns appear to violate core RDS assumptions ^5, 12^.

The prevalence of wheelchair users has rapidly increased over the last half century due, in part, to advancing medical care, ageing populations, increasing community supports, increased prescription of wheelchairs, and changes in attitudes to disablement such that people may feel less stigmatised about using a wheelchair ^13, 14^. Despite this, robust epidemiological research with this group in New Zealand and Australia is scant ^15, 16^. Contacting wheelchair users in the community is challenging. Recruitment approaches are often limited to using disability organisations and personal contacts, which can differentially exclude many wheelchair users ^17^. Consequently, wheelchair users may constitute a ‘hidden population’, under-researched and excluded from population estimates ^18^. Furthermore, many countries, including New Zealand, have yet to establish registries of wheelchair users which could provide a reliable sampling frame ^16, 18^.

Here we report our experience of applying a RDS methodology to a survey of wheelchair users in New Zealand. To our knowledge this is the first time RDS has been applied to people who use wheelchairs, and could potentially offer a significant new sampling approach in epidemiology and disability fields.

## Methods

After a successful pilot with wheelchair users, this study employed a short internet-based national survey which was open from October 2015 until January 2016. Administered through the SurveyMonkey™ website, an information sheet and video were embedded within the survey preamble (see Supplementary material). The information sheet stated that informed consent was implied through the voluntary participation in the survey. Ethics approval was obtained from the University of Canterbury Human Ethics Committee (reference HEC 2015/117). Eligibility criteria included: wheelchair use as the primary form of mobility; being a New Zealand resident; aged 16 years or more; being able to read English; having internet access; and, having an operational email account.

Invitations seeking ‘seed’ participants were circulated to various national disability organisations serving members with a range of impairments that lead to wheelchair use. People expressing interest in being seeds contacted the researcher, who confirmed eligibility and then sent a recruitment code and a link to the survey website. Once a participant completed the survey, they were thanked and emailed three unique recruitment codes. Participants were asked to recruit a maximum of three other wheelchair users, following Heckathorn’s (1997) recommendations ^2^. This limit of three was premised on two primary reasons: to ensure that a broad array of participants are recruited; and, to prevent the emergence of semi-professional recruiters. Participants were asked to email one code and the survey link to three other persons they knew who were likely to satisfy the eligibility criteria. This process was envisaged to continue for multiple recruitment waves. Participation was incentivised (an entry into a draw to win an iPad); one entry for completing the survey, and another when each person they recruited completed the survey. Recruitment chains were tracked through tracing the recruitment codes. A predetermined reminder protocol was triggered when seeds or their peers failed to respond.

## Results

Twenty wheelchair users expressed interest in participating as seeds, of whom 12 completed the survey (60% response rate). All 12 seeds were asked to recruit a maximum of three wheelchair users. Only four seeds were successful in recruiting further participants (three seeds each recruited two participants, and one seed recruited one participant), accumulating in a total of seven first wave participants. Despite all seven first wave participants being asked to recruit a maximum of three wheelchair users, using a clearly stated invitation and reminder protocol, no second wave participants completed the survey. Thus, the final sample was composed of 19 wheelchair users. Mean age of participants was 55.6 years (range: 28–73 years), and nine were women. Survey completion time ranged between 7 and 36 minutes.

Our recruited sample of 19 wheelchair users, however, failed to satisfy the requirements needed to reach equilibrium; the point at which the sample composition becomes independent of the initial seeds, thereby enabling the calculation of unbiased population estimates ^4^. This failure stems from the study’s premature termination, where only one measurement wave was completed. Even in the best-case scenario where equilibrium is reached in the smallest possible number of waves, namely one, no useable data could be produced after equilibrium. Furthermore, when only a single wave is conducted, all participants are within a single degree of separation from the seed participants accessed by the researcher. Such a sample lacks what Heckathorn terms ‘sociometric depth’ and it would, in all likelihood, fail to be representative of the entire hidden population ^10^. For these reasons, an empirical assessment of equilibrium was not formally undertaken here, as it was both conceptually and statistically impossible for the data captured within this study to be in-equilibrium.

## Discussion

Despite a rigorous recruitment process and offering incentivising participation, our use of RDS failed as an effective sampling approach amongst wheelchair users in New Zealand. There are a number of possible explanations as to why this occurred. The target population of the study was novel compared with hidden populations generally targeted by RDS studies. Research using RDS typically samples stigmatised populations, such as those with greater risk of HIV, including injecting drug users ^5^, men who have sex with men ^12, 19^, and sex workers ^20^. Wheelchair users have experienced increased integration into many societies in recent years and are arguably less stigmatised when compared to populations traditionally sampled using RDS. Populations experiencing greater stigma may have a tendency to establish stronger social and internal networks, helping to facilitate the RDS requirement that the population being sampled has sufficiently strong internal networks which enable the random recruitment of other members of the population. With no literature to our knowledge regarding the internal networks of wheelchair users, it is unknown whether wheelchair users would satisfy the random recruitment criteria of RDS. Although the precise mechanism by which perceived stigma might affect RDS participation is unknown it, nonetheless, remains noteworthy. Second, the use of an unguaranteed reward (entry into a draw for an iPad) for survey completion has not been previously reported in RDS studies. This lack of guaranteed reward may have influenced participation. In addition, RDS studies often offer participants additional non-monetary free services related to the mitigation of HIV risk through counselling and educational material ^7^.

Exploring the areas of network characteristics, acceptability of RDS, appropriate incentive options, and seed selection have all been suggested as important for assessing the feasibility and appropriateness of RDS in certain populations ^20^. Here, critical feedback on the appropriateness of the incentives, RDS methodology, elicitation mechanism and platform, and the survey itself was obtained from the pilot group – but not from the seeds. Formative research regarding specific seed selection is warranted. First, we recommend judiciously selecting diverse seeds who have large social networks, which should facilitate positive growth of recruitment chains. This increases the chances of participants with diverse characteristics being recruited and helping to avoid the exclusion of isolated subpopulations and individuals. It also helps to increase the speed at which sampling equilibrium can be reached ^9^. Indeed, one RDS study exploring people who inject drugs in Sydney Australia reported that 80% of their participants resulted from one seed ^21^. Second, meeting with seeds to provide greater education regarding goals and protocol of the survey might have improved the recruitment rates of our survey. Providing greater education to seeds might have increased their commitment to the goals of the survey, increasing the chances that seeds will report favourably about the survey, accurately explain the survey goals, and be motivated to pass on all three recruitment codes ^9^. Despite the traditionally low response rates and impersonal nature of electronic surveys ^1^, administering surveys electronically is becoming more feasible and successful with populations who use wheelchairs ^22^, and having informed and enthusiastic seeds might have encouraged greater response rates. Until such time as these and other factors, and their implications for recruitment, are better understood we feel that using RDS for recruiting wheelchairs users may have limited merit, and recommend formative research to optimise success.

## Conclusions

Wheelchair users are an increasingly prevalent population in society who often lack an adequate sampling frame, and sampling approaches enabling valid population based estimates are becoming increasingly necessary. This paper reported the failure of RDS to survey wheelchair users. Despite the unsuccessful recruitment in this study, further research exploring the application of RDS with wheelchair users is recommended before discounting this sampling approach in this population.

## Data availability

The data referenced by this article are under copyright with the following copyright statement: Copyright: © 2016 Bourke JA et al.

Data are available upon request from the corresponding author to protect participant identity. Demographic data will be pooled to protect participant identity, as individual-level demographic data could be theoretically traceable due to the small sample size, and suspected small national population of wheelchair users.

## Consent

All participants were informed that the voluntary completion of the survey implied informed consent, including for the publication of survey data.

## Supplementary material

Internet-based national survey.

.

Information sheet.
