# Supplementary material for: Respondent driven sampling of wheelchair users: A lack of traction?
Source: F1000Res. 2016 Aug 26;5:753. Originally published 2016 Apr 26. [Version 2] doi: 10.12688/f1000research.8605.2 (PMC5017286; doi:10.12688/f1000research.8605.2)
Supplement: Supplementary file 1 [file f1000research-5-10247-s0000.tgz › a643151c-ad8e-48ef-8fec-3b1ab0b8c915.pdf]

Introduction

Thank you for agreeing to take part in this survey to find out about how wheelchair users have experienced community inclusion in the five years following the September 2010 Canterbury earthquake. This information can help local authorities understand the important factors for wheelchair users as communities rebuild.

Although this survey is framed around the Christchurch earthquakes, we are interested in hearing from people who live in all parts of New Zealand. This will help us to understand any differences in the experience of community inclusion between wheelchair users who live in in Christchurch, and those who live in communities outside of Christchurch. The survey should take around 20 minutes to complete. You can read a detailed information sheet by clicking here. You can also view and information video by clicking here. When you complete the survey you will automatically enter the draw to win an iPad (funded by the University of Canterbury PhD student research fund allocation. Prize drawn in 2016).

Once you complete the survey we would really appreciate your help to find 3 other people to complete this survey. When you finish the survey you will be automatically emailed 3 codes along with instructions on how to email one code to each person so they can complete the survey. You get one entry into the iPad draw for completing the survey yourself. You can also get up to three more entries if those you invite to take part also complete the survey – total of 4 entries in all.

Your participation is completely voluntary. If you require any assistance to complete the survey please ask someone you trust to help you. If you have any questions about the survey please contact John Bourke at john.bourke@pg.canterbury.ac.nz

If you have any concerns please contact the University of Canterbury's Human Ethics Committee, via human-ethics@canterbury.ac.nz

1. Please enter your recruitment code (sent to you in the recruitment email):

2. Please enter your email address (to receive codes to send out to three others. We will contact you by email if you win the ipad competition):

3. Approximately how many other wheelchair users do you know? (Enter number):

Section 1: Background Information

Background information helps us to find out how much the survey sample is like the general population of New Zealand.

4. Please enter your email address (to contact you if you win the ipad competition):

5. On what date were you born? (enter dd/mm/yyyy e.g. 04/06/1989)

6. What is your gender? (select one)

- Male
- Female
- Gender Diverse

7. Which ethnic group do you belong to? (select all that apply)

- NZ European/Pakeha
- NZ Māori
- Pacific
- Asian
- Indian
- Prefer not to say
- Other (please specify)

8. Which best describes your household’s annual income before tax?

- Loss
- No income
- Less than $30,000
- $30,001-$60,000
- $60,001-$100,000
- More than $100,000
- Don't know
- Prefer not to say

9. Which of the following best describes your current usual housing situation:

- Home owner
- Renting
- Rehabilitation unit
- Long-term care facility
- Temporary accommodation
- Other (please specify)

10. Which of the following best describes your usual housing situationat the time of the September 2010 earthquake:

- Home owner
- Renting
- Rehabilitation unit
- Long-term care facility
- Temporary accommodation
- Other (please specify)

11. Which best describes your current usual living arrangement:

- ○ Alone
- ○ With spouse/partner, no children
- ○ With spouse/partner and your child(ren) or step-child(ren)
- ○ With child(ren) or step-child(ren), but no other adult(s)
- ○ With other family relatives
- ○ With non-relatives
- ○ Other (please specify)

12. Which best describes your usual living arrangementat the time of the September 2010 earthquake

- ○ Alone
- ○ With spouse/partner, no children
- ○ With spouse/partner and your child(ren) or step-child(ren)
- ○ With child(ren) or step-child(ren), but no other adult(s)
- ○ With other family relatives
- ○ With non-relatives
- ○ Other (please specify)

13. Which area do you currently usually live in?

- ○ Christchurch (red-zone)
- ○ Christchurch (elsewhere)
- ○ Other region in New Zealand (please specify)

14. Which area did you usually live in at the time of the September 2010 earthquake?

- ○ Christchurch (red-zone)
- ○ Christchurch (elsewhere)
- ○ Other region in New Zealand (please specify)

15. What impact did the 2010/2011 Christchurch earthquakes have on your usual housing situation?

- ○ None
- ○ A small amount (e.g. minor house damage)
- ○ A moderate amount (e.g. had to move temporarily)
- ○ A significant amount (e.g. permanent relocation)
- ○ Other (please specify)

Section 2: Mobility Information

Answers to these questions will help us understand your wheelchair use.

16. What is your current usual primary mode of mobility?

- ○ A manual wheelchair
- ○ A power wheelchair
- ○ A mix of manual and power wheelchairs
- ○ Other (please specify)

[ ]

17. What was your usual primary mode of mobility at the time of the September 2010 earthquake?

- ○ A manual wheelchair
- ○ A power wheelchair
- ○ A mix of manual and power wheelchairs
- ○ Other (please specify)

[ ]

18. In what year did you begin using a wheelchair as your usual primary mode of mobility? (e.g. 1996):

[ ]

19. Which best describes the impairment that requires you to use a wheelchair? (select one)

- ○ Spinal Cord Injury
- ○ Cerebral Palsy
- ○ Multiple Sclerosis
- ○ Muscular Dystrophy
- ○ Stroke
- ○ Amputation
- ○ Traumatic Brain Injury
- ○ Other (please specify)

[ ]

20. Do you receive home help and/or attendant care support? (e.g. for personal cares, driving, or housework)

- Yes
- No

21. If you answered Yes to Q19, approximately how many hours per day:

- 0-4
- 5-9
- 10-14
- 15-19
- 20-24

22. Do you receive financial support because of your impairment? (e.g. for care, equipment etc.)

- Yes
- No

23. If you answered Yes to Q21, who is the primary provider of that funding? (select one):

- ACC
- Ministry of Health
- Other (please specify)

Section 3: Earthquake Experience

This section asks about your earthquake exposure and experience.

24. Were you present in the Christchurch district during any of the following earthquakes? (select all that apply):

- ☐ September 4th 2010
- ☐ February 22nd 2011
- ☐ June 13th 2011
- ☐ December 22nd 2011

25. About how much time have you spent living in Christchurch in the 5 years since September in 2010?

- ◯ None or nearly none
- ◯ Some (long periods of time away – equal to or more than 2 years away)
- ◯ Most of the time (short periods of time away – less than 2 years away)
- ◯ The whole time or almost the whole time

26. Is Christchurch your primary place of residence?

- ◯ Yes
- ◯ No

27. If you answered Yes to Q25, was Christchurch your primary place of residence before September 2010?

- ◯ Yes
- ◯ No

28. If you answered No to Q26, in which month/year did Christchurch become your primary place of residence? (enter mm/yyyy e.g. 07/2011)

[ ]

29. Would you say that your overall quality of life is (select one):

- ◯ Extremely poor
- ◯ Poor
- ◯ Neither poor nor good
- ◯ Good
- ◯ Extremely good

30. Since September 2010 would you say your quality of life has (select one):

- ◯ Decreased significantly
- ◯ Decreased to some extent
- ◯ Stayed about the same
- ◯ Increased to some extent
- ◯ Increased significantly

Section 4: Community Access

This section asks about your experience of community accessibility over the last four years. Please select one response for every type of access.

31. In the last 6 months, how hard was it for you to:

	Impossible	Always hard	Often hard	Occasionally hard	Never hard	Not applicable
Access footpaths due to uneven surfaces	◯	◯	◯	◯	◯	◯
Access footpaths due to repair and construction work	◯	◯	◯	◯	◯	◯
Find suitable accessible housing	◯	◯	◯	◯	◯	◯
Find accessible places for social interactions	◯	◯	◯	◯	◯	◯
Access your workplace	◯	◯	◯	◯	◯	◯
Access your education centre	◯	◯	◯	◯	◯	◯
Receive attendant care services	◯	◯	◯	◯	◯	◯
Find accessible car parks	◯	◯	◯	◯	◯	◯
Gain entry to, and move inside buildings built before the earthquakes	◯	◯	◯	◯	◯	◯
Gain entry to, and move inside buildings built after the earthquakes	◯	◯	◯	◯	◯	◯
Access public spaces in your community	◯	◯	◯	◯	◯	◯

32. In the 6 months immediately after the time of the September 2010 earthquake how hard was it for you to:

	Impossible	Always hard	Often hard	Occasionally hard	Never hard	Not applicable
Access footpaths due to uneven surfaces	○	○	○	○	○	○
Access footpaths due to repair and construction work	○	○	○	○	○	○
Find suitable accessible housing	○	○	○	○	○	○
Find accessible places for social interactions	○	○	○	○	○	○
Access your workplace	○	○	○	○	○	○
Access your education centre	○	○	○	○	○	○
Receive attendant care services	○	○	○	○	○	○
Find accessible car parks	○	○	○	○	○	○
Gain entry to, and move inside buildings built before the earthquakes	○	○	○	○	○	○
Gain entry to, and move inside buildings built after the earthquakes	○	○	○	○	○	○
Access public spaces in your community	○	○	○	○	○	○

33. In the 6 months immediately before the time of the September 2010 earthquake how hard was it for you to:

	Impossible	Always hard	Often hard	Occasionally hard	Never hard	Not applicable
Access footpaths due to uneven surfaces	○	○	○	○	○	○
Access footpaths due to repair and construction work	○	○	○	○	○	○
Find suitable accessible housing	○	○	○	○	○	○
Find accessible places for social interactions	○	○	○	○	○	○
Access your workplace	○	○	○	○	○	○
Access your education centre	○	○	○	○	○	○
Receive attendant care services	○	○	○	○	○	○
Find accessible car parks	○	○	○	○	○	○
Gain entry to, and move inside, buildings	○	○	○	○	○	○
Access public spaces in your community	○	○	○	○	○	○

34. If access has changed in the last 6 months, compared to the 6 months before the time of the September 2010 earthquake, is this because of (select all that apply):

- ☐ The Canterbury earthquakes
- ☐ A significant change in your health status
- ☐ Housing relocation
- ☐ Employment relocation
- ☐ A significant change in family/carer support
- ☐ A significant change to funding or finances
- ☐ Other (please specify)

[ ]

Section 5: Social Networks

This section asks about your social networks over the last four years. The term 'sense of community' refers to how included you feel with the people and in the places in your neighbourhood.

35. Thinking about the last 6 months, to what extent do you agree with the following statement?
"I feel a sense of community with others in my neighbourhood"

- ◯ Strongly disagree
- ◯ Disagree
- ◯ Neither disagree or agree
- ◯ Agree
- ◯ Strongly agree

36. How does this current sense of community with others in your neighbourhood in the last 6 months compare with what it was like in the 6 months after the time of the September 2010 earthquake?

- ◯ A decreased sense of community with others
- ◯ No different
- ◯ An increased sense of community with others

37. How does this current sense of community with others in your neighbourhood in the last 6 months compare with what it was like in the 6 months before the time of the September 2010 earthquake?

- ◯ A decreased sense of community with others
- ◯ No different
- ◯ An increased sense of community with others

38. If you were faced with another serious illness or injury, or needed emotional support during a difficult time, is there someone you could turn to for help?

- ◯ Yes
- ◯ No
- ◯ Not sure

39. If you answered Yes to Q37, who would you turn to for help? (select all that apply)

- [ ] Family
- [ ] Friends
- [ ] Faith-based group
- [ ] Cultural group
- [ ] Neighbourhood group
- [ ] Clubs and societies
- [ ] Health or social support worker
- [ ] Parenting networks
- [ ] Work colleagues
- [ ] Online community
- [ ] Rūnanga
- [ ] Other (please specify)

Section 6: The Future

This section asks your opinion about accessibility in your community in the future. The term ‘accessibility’ refers to the ability for you to take part in your community e.g. having the ability to access places and interact with the people that are important to you.

40. Select the option that you think best answers the following statement:
"I feel that since September 2010, the opportunity to improve accessibility in my community is..."

- ◯ Non-existent
- ◯ Very low
- ◯ Low
- ◯ Medium
- ◯ High
- ◯ Very high

41. Since the time of the September 2010 earthquakes, do you feel the opportunity to improve accessibility in your community has:

- ◯ Decreased
- ◯ Not changed
- ◯ Increased

42. To what extent do you feel confident that:

	Not at all confident	Not very confident	Neutral	Confident	Very confident	Don't know
Central government (e.g. CERA) is making earthquake recovery decisions that are in the best interests of rebuilding an accessible city?	○	○	○	○	○	○
Local council is making earthquake recovery decisions that are in the best interests of rebuilding an accessible city?	○	○	○	○	○	○
Building developers are making earthquake recovery decisions that are in the best interests of rebuilding an accessible city?	○	○	○	○	○	○

43. All things considered, what impact has the Christchurch sequence of earthquakes had on you? (select one):

- ○ None
- ○ Some impact
- ○ Moderate impact
- ○ Significant impact

44. Do you have any other comments you would like to makeabout community inclusion following the Canterbury earthquakes?
