# Supplementary material for: Respondent driven sampling of wheelchair users: A lack of traction?
Source: F1000Res. 2016 Aug 26;5:753. Originally published 2016 Apr 26. [Version 2] doi: 10.12688/f1000research.8605.2 (PMC5017286; doi:10.12688/f1000research.8605.2)
Supplement: Supplementary file 2 [file f1000research-5-10247-s0001.tgz › c652648c-bbc4-4a34-afb5-9e44c6d267ba.docx]

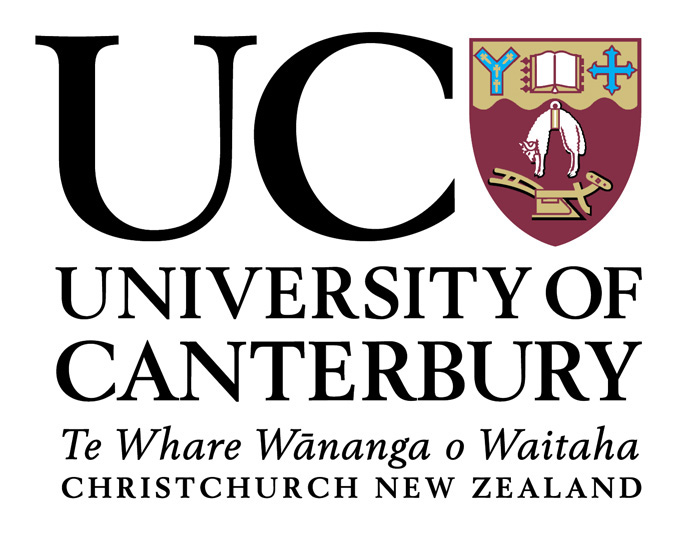


Rebuilding following the Canterbury earthquakes: Wheelchair users experience of community inclusion.

**INFORMATION SHEET FOR PARTICIPANTS**

This survey seeks to understand how people who use wheelchairs have experienced community inclusion in the four years following the 2010/11 Canterbury earthquakes. This information sheet tells you who is conducting the study, the type of participants we are hoping to recruit, and what is involved if you do choose to take part. Your participation is completely voluntary. Please take the time to read through the information and feel free to contact one of the investigators for more information before making a decision (contact details are at the bottom of the last page).

**Who is doing the study?**

Hi there, my name is John Bourke and I am the principal investigator for this study. This study forms part of my doctoral research at the University of Canterbury. I have been using a power wheelchair as my main form of mobility after having a spinal cord injury in 2005. My supervisors for this project are Professor Philip Schluter (University of Canterbury), Associate Professor Jean Hay-Smith (University of Otago) and Dr Deborah Snell (Burwood Academy of Independent Living).

**Aim of the survey**

For this study I aim to survey people who use wheelchairs regarding their experience of community inclusion in the four years following the 2010/11 Canterbury earthquakes. Simply put, community inclusion means being able to interact with the people and in the places that are important to you. This information can help local authorities understand the important factors for wheelchair users as communities rebuild.

**What types of participants are required?**

I am interested in surveying people who use a wheelchair as their main form of mobility, reside in New Zealand, are 16 years or older, are able to read English, and have an operational email account and access to the internet.

**What will you be asked to do?**

If you agree to take part in the survey, follow the link in this email which will take you to the survey on the Survey Monkey™ website. The survey should take around 20 to 30 minutes to complete. Once you complete the survey we would really appreciate your help to find 3 other people to complete this survey. To do this I will send you three codes along with instructions on how to email one code to each person so they can complete the survey. This type of survey and recruitment is called a Respondent Driven Survey approach.

**Benefits of the study**

When you complete the survey you will automatically enter the draw to win an iPad (funded by the University of Canterbury PhD student research fund allocation). You get one entry into the draw for completing the survey yourself. You can also get up to three more entries if those you invite to take part also complete the survey – total of 4 entries in all.

Although there are no other direct benefits from taking part, we also hope that the results of this study will highlight factors important to people who use wheelchairs as communities rebuild following natural disasters such as the 2010/11 Canterbury earthquakes.

**What will happen to the information collected?**

All information you enter into the survey will be kept private and secure. Your email address will only be known by myself and kept on a password-protected computer. All other information you submit will be converted into numbers so that anyone else needing to use the data, such as a statistician, will not be able to identify you. No identifying data will be presented in my doctoral thesis or any other research outcomes from this survey. The information you enter into the survey will not be passed on to any other parties or used for any other purpose apart from this study. The data will be kept in a locked cabinet for 10 years before being destroyed. This is standard practice for research.

**Risks of the study**

We understand that the survey will ask you about aspects relating to the 2010/11 Canterbury earthquakes and there is a chance this might be upsetting. You can stop the survey at any time. If you become concerned with any aspect of this study, or the subject of the earthquakes causes any distress, the following organisations can provide advocacy and assistance:

Health and Disability Services Consumer Advocate

The Health and Disability Advocacy can assist you with making sure your rights are respected. An advocate can listen to your concerns, provide information on your rights, and provide practical support regarding your options. To contact a Health and Disability Services Consumer Advocate, free phone 0800 555 050 for a list of free advocates in your area, or check <http://advocacy.hdc.org.nz/find-an-advocate.aspx>).

The Canterbury Earthquake Support Line

Canterbury Earthquake Support is a free and confidential service designed to support people following the Canterbury earthquakes. A coordinator can provide you with information and connect you with a wide range of services that offer practical information, support, and advice. You can contact the Canterbury earthquake Support Line on 0800 777 846

The University of Canterbury Human Ethics Committee

The University of Canterbury Human Ethics Committee can help with any ethical concerns regarding the conduct of the study. Please address any concerns to the University of Canterbury’s Human Ethics Committee via [human-ethics@canterbury.ac.nz](mailto:human-ethics@canterbury.ac.nz)

If taking part in the survey brings up any distressing issues and you would like more support, one of my co-investigators (Dr Deborah Snell) is a registered clinical psychologist and would be happy to provide you with information about where to go to get help. Her contact information is provided below.

**Where can I get more information about the study?**

John Bourke

PhD Candidate

School of Health Sciences

University of Canterbury

[john.bourke@pg.canterbury.nz](mailto:Boujo725@student.otago.ac.nz)

(03) 364 2987 extension 4128

Professor Philip Schluter

School of Health Sciences

University of Canterbury

[philip.schluter@canterbury.ac.nz](mailto:philip.schluter@canterbury.ac.nz)

(03) 364 2987 extension 44357

Associate Professor Jean Hay-Smith

Rehabilitation Teaching and Research Unit,

University of Otago

[jean.hay-smith@otago.ac.nz](mailto:jean.hay-smith@otago.ac.nz)

(03) 474 0999

Dr Deborah Snell

Academic Director

Burwood Academy of Independent Living (BAIL)

Private Bag 4708

Christchurch 8140

[debbie.snell@burwood.org.nz](mailto:debbie.snell@burwood.org.nz)

(03) 383 6871
