# Supplementary material for: Respondent driven sampling of wheelchair users: A lack of traction?
Source: F1000Res. 2016 Aug 26;5:753. Originally published 2016 Apr 26. [Version 2] doi: 10.12688/f1000research.8605.2 (PMC5017286; doi:10.12688/f1000research.8605.2)
Supplement: Peer review history

# Peer review: Referee response for version 2

**DOI:** 10.5256/f1000research.10247.r15906

**Published:** 2016-09-22

**Authors:** Jesse Kokaua

**Affiliations:** 1 Pacific Islands Research & Student Support Unit, University of Otago, Dunein, New Zealand

The changes the authors have made have addressed the recommendations I had made for the first version.

I have read this submission. I believe that I have an appropriate level of expertise to confirm that it is of an acceptable scientific standard.

# Peer review: Referee response for version 2

**DOI:** 10.5256/f1000research.10247.r16242

**Published:** 2016-09-12

**Authors:** A James O'Malley

**Affiliations:** 1 Department of Biomedical Data Science and The Dartmouth Institute of Health Policy and Clinical Practice, Geisel School of Medicine at Dartmouth, Lebanon, NH, USA

I have read the revised article and feel that the authors have responded to my concerns as well as possible given the space limitations. As such, I am willing to change my decision to Approved.

I have read this submission. I believe that I have an appropriate level of expertise to confirm that it is of an acceptable scientific standard.

# Peer review: Referee response for version 2

**DOI:** 10.5256/f1000research.10247.r16076

**Published:** 2016-09-05

**Authors:** John F Smith

**Affiliations:** 1 Postgraduate Tropical Medicine Program, Faculty of Medicine, Kohn Kaen University, Khon Kaen, Thailand

I agree with the revisions, and therefore change my recommendation to Approved.

I have read this submission. I believe that I have an appropriate level of expertise to confirm that it is of an acceptable scientific standard.

# Peer review: Referee response for version 1

**DOI:** 10.5256/f1000research.9259.r14896

**Published:** 2016-07-11

**Authors:** A James O'Malley

**Affiliations:** 1 Department of Biomedical Data Science and The Dartmouth Institute of Health Policy and Clinical Practice, Geisel School of Medicine at Dartmouth, Lebanon, NH, USA

***Title and Abstract***: The title is catchy and appropriate given the content of the article.

***Article content*** *:* The article is very well written and is easy to read and follow. The article would be much improved if it included an introduction to respondent drive sampling (RDS) with particular emphasis on the assumptions required for RDS to work (reaching equilibria and otherwise) written in clear terms to a lay audience. Describe what it means for the sampling process to be in equilibrium. Currently, readers do not have the necessary information to reach an informed conclusion as to why RDS did not work well in this instance or to assess whether it is applicable to their own work.

 The rationale for RDS assumes the existence of a social network in which the probability of a tie between a wheelchair user and a randomly selected wheelchair user exceeds the probability of a tie between a wheelchair user and a nonuser wheelchair user. What evidence that this is true? Do wheelchair users have sufficient social and other relationships importantly enough for them to be able to name sufficient other wheelchair users for the next round of sampling. Is it possible that many wheelchair users are isolates (in the network sense) because they are highly functional in everyday life and thus have reduced need for a community of wheelchair users? I don’t know the answers to these questions and still don’t as the needed background information is not provided in the article.

 It would have been helpful to have been told the current state of knowledge about social networks among wheelchair users along with a connection of such knowledge to the assumptions under which RDS can yield unbiased population-level inferences. I would like to see some discussion of how RDS handles scenarios such as isolates in the wheelchair user network. Presumably, the only way such individuals make it into the sample is if they were randomly selected in the first (i.e., seed) wave.

 Please add some general intuition for how RDS yields unbiased population-level estimates. Discuss the role of and derivation of individuals’ sampling probabilities and thus sampling weights for computing population estimates. For example, the work of Thompson (2006a, 2006b, 2003, 2000 – see below – the 2012 text on Sampling and recent arXiv contributions) should be reviewed and used to inform the article.

 References:

1. Thompson, Steven K. Targeted random walk designs. Survey Methodology 2006. 32:11-24
2. Thompson, Steven K. Adaptive web sampling. Biometrics 2006. 62:1224-1234
3. Chow, Mosuk, Thompson, Steven K. Estimation with link-tracing sampling designs - A Bayesian approach. Survey Methodology 2003. 29:197-205
4. Thompson, Steven K., Frank, Ove. Model-based estimation with link-tracing sampling designs. Survey Methodology 2000. 26:87-98

 

***Data (if applicable)***: Details about the design/data for the study are lacking. It is stated that 20 wheelchair users were the initial seeds of which 12 agreed to participate (60% response rate). It then appears as though 4 of these 12 participants culled 7 further participants in the second stage of sampling. Question: Did the remaining 8 seeds not generate any wheelchair users (i.e., does the enrolment rate for the second stage equal 7/12) or was the plan to only ask 33% of the initial seeds for links to trace (in which case the enrolment rate is 7/4)? In the third stage, there was no addition to the sample-size. Were the 7 participants who entered at the second stage each asked to name other wheelchair users; is the enrolment rate at the third stage 0/7 or 0/x, where x<7? Was there any limit as to how many wheelchair users a seed to subsequently enrolled person could name? A lot of these questions would be answerable if the design of the RDS was described in the article!

***Conclusions*** *:* The discussion makes a number of good points that have appear sound and logical. The primary point made is that the current study may have failed because the wheelchair user population has different characteristics from populations where RDS has been used successfully. This is a valid point. But shouldn’t this point have been considered when the study was design? Was there any prior information to inform the parameters of the RDS design? Maybe RDS would work quite well for the wheelchair user population as long as the number of initial seeds is much greater than 20. A more positively-framed Discussion might leave readers with a more balanced appreciation for using RDS in their own studies of wheelchair users or other new populations.

I have read this submission. I believe that I have an appropriate level of expertise to confirm that it is of an acceptable scientific standard, however I have significant reservations, as outlined above.

# Response

**Published:** 2016-08-23

**Authors:** Philip Schluter

**Affiliations:** University of Canterbury

Thank you greatly for your prompt, encouraging, and constructive feedback on our paper. Together with the comments made by the other two reviewers, your feedback has resulted in what we feel is a much improved paper.

 We would like to take this opportunity to make two notes. [1] The original submission was restricted to 1000 words in total – but, with permission from the journal, this constraint has been relaxed and we are able to include more material. This means we are able to broaden discussion of relevant issues and thus strengthen the manuscript. [2] This paper was submitted to the journal as an ‘observation article’, defined as: *Observation Articles allow the description of a novel observation that may be unexpected, and possibly currently without explanation. An observation can be a phenomenon that has been identified in field work, in the laboratory or through experimental analysis* (see: http://f1000research.com/for-authors/article-guidelines/observation-articles).

***Title and Abstract*** *: The title is catchy and appropriate given the content of the article.*

 Thank you for the positive comment.

***Article content*** *:* *The article is very well written and is easy to read and follow. The article would be much improved if it included an introduction to respondent drive sampling (RDS) with particular emphasis on the assumptions required for RDS to work (reaching equilibria and otherwise) written in clear terms to a lay audience. Describe what it means for the sampling process to be in equilibrium. Currently, readers do not have the necessary information to reach an informed conclusion as to why RDS did not work well in this instance or to assess whether it is applicable to their own work.*

 We thank the reviewer for describing our article as well written and easy to read and follow. We agree that an introduction to RDS would improve the article. Our original submission was limited by word restrictions and article scope, as outlined above. However, in this revision, we have included more details. Specifically, we have included text in the Introduction section (see second paragraph) providing a brief introduction to RDS, and describing equilibrium (see third paragraph). We have expanded the Results section (second paragraph) to describe why equilibrium was not reached in our study.

*The rationale for RDS assumes the existence of a social network in which the probability of a tie between a wheelchair user and a randomly selected wheelchair user exceeds the probability of a tie between a wheelchair user and a nonuser wheelchair user. What evidence that this is true?*

 We had no evidence to suggest this was true or false and felt on balance that the use of RDS in our study was warranted as there was a reasonable enough chance that it could be true without evidence to the contrary. Conducting a revised search on PubMed, Google scholar, and Science Direct, we could find no literature explicitly addressing this issue (we have included a sentence in the first paragraph of the Discussion section describing this).

*Do wheelchair users have sufficient social and other relationships importantly enough for them to be able to name sufficient other wheelchair users for the next round of sampling. Is it possible that many wheelchair users are isolates (in the network sense) because they are highly functional in everyday life and thus have reduced need for a community of wheelchair users? I don’t know the answers to these questions and still don’t as the needed background information is not provided in the article.*

 This is indeed possible. The fact that a person uses a wheelchair does by no means infer that they know other people who use wheelchairs, let alone have strong internal networks with other wheelchair users. With respect to these questions we have included new text in the Discussion section (see first paragraph) regarding stigma and whether or not wheelchair users have sufficiently strong internal networks that enable random recruitment of other wheelchair users.

*It would have been helpful to have been told the current state of knowledge about social networks among wheelchair users along with a connection of such knowledge to the assumptions under which RDS can yield unbiased population-level inferences. I would like to see some discussion of how RDS handles scenarios such as isolates in the wheelchair user network. Presumably, the only way such individuals make it into the sample is if they were randomly selected in the first (i.e., seed) wave.*

 As noted above, the current state of knowledge regarding the social networks among wheelchair users is unknown. As a result we cannot know if some wheelchair users are isolates. RDS theory assumes that the population being sampled comprises of a complete social network component, so in theory every person within their population has a probability of being sampled. In practice, to overcome isolated subpopulations, it is recommended that seeds from diverse subpopulations be selected. We have included a sentence to this effect in the second paragraph of the Discussion section.

*Please add some general intuition for how RDS yields unbiased population-level estimates. Discuss the role of and derivation of individuals’ sampling probabilities and thus sampling weights for computing population estimates.*

 Due to original word count, we had not included information clarifying that question two of our survey asked participants to provide an estimate of their network size (a requirement of RDS). Had our study produced in-equilibrium data (see new text in second paragraph in Introduction section, and new text in second paragraph of the Results section), sampling weights would have been allocated accordingly with those with smaller social network sizes being given a higher weight.

*For example, the work of Thompson (2006a, 2006b, 2003, 2000 – see below – the 2012 text on Sampling and recent arXiv contributions) should be reviewed and used to inform the article.*

*References:*

1. *Thompson, Steven K. Targeted random walk designs. Survey Methodology 2006. 32:11-24.*
2. *Thompson, Steven K. Adaptive web sampling. Biometrics 2006. 62:1224-1234.*
3. *Chow, Mosuk, Thompson, Steven K. Estimation with link-tracing sampling designs - A Bayesian approach. Survey Methodology 2003. 29:197-205.*
4. *Thompson, Steven K., Frank, Ove. Model-based estimation with link-tracing sampling designs. Survey Methodology 2000. 26:87-98.*

 

 Thank you greatly for suggesting these references, which were reviewed and indeed helped with our thinking. Because our study failed to produce in-equilibrium data that warranted further analysis and consideration of sampling weights and sampling probabilities, substantial engagement with the topics in these papers was felt to be beyond the scope of our paper.

***Data (if applicable)*** *: Details about the design/data for the study are lacking. It is stated that 20 wheelchair users were the initial seeds of which 12 agreed to participate (60% response rate). It then appears as though 4 of these 12 participants culled 7 further participants in the second stage of sampling. Question: Did the remaining 8 seeds not generate any wheelchair users (i.e., does the enrolment rate for the second stage equal 7/12) or was the plan to only ask 33% of the initial seeds for links to trace (in which case the enrolment rate is 7/4)*? 

 All 12 seeds were asked to recruit 3 peers and for links to trace. Despite this, 8 seeds did not generate any more participants. The enrolment rate was 7/12. To clarify recruitment/response rates, text has been included in the first paragraph of the Results section.

*In the third stage, there was no addition to the sample-size. Were the 7 participants who entered at the second stage each asked to name other wheelchair users; is the enrolment rate at the third stage 0/7 or 0/x, where x<7?*

 The enrolment rate was 0/7. All 7 participants were asked to recruit 3 peers and for links to trace, but no participants were recruited. To clarify recruitment/response rates, text has been included in the first paragraph of the Results section.

*Was there any limit as to how many wheelchair users a seed to subsequently enrolled person could name? A lot of these questions would be answerable if the design of the RDS was described in the article!*

 Thank you for raising this issue. All seeds and subsequent participants were asked to recruit a maximum of 3 peers and for links to trace. Heckathorn (1997) recommends that each respondent is limited to 3 ID codes to pass on to others, to ensure that a broad array of participants is recruited, and to prevent the emergence of semi-professional recruiters. We have now included two sentences describing this in the second paragraph in the Methods section.

***Conclusions*** *:* *The discussion makes a number of good points that have appear sound and logical. The primary point made is that the current study may have failed because the wheelchair user population has different characteristics from populations where RDS has been used successfully. This is a valid point. But shouldn’t this point have been considered when the study was designed?*

 Yes, a valid point. In retrospect the potential impact of this point could have attracted greater consideration. However, we had no evidence to suggest it would be detrimental to the success of our employment of RDS. We also acknowledged that our application of RDS with wheelchair users was unprecedented and novel, and many outcomes were simply unknown. In the absence of any reliable sampling frame or registry, we believed the potential success amongst this population (on balance) was greater than the risks.

*Was there any prior information to inform the parameters of the RDS design? Maybe RDS would work quite well for the wheelchair user population as long as the number of initial seeds is much greater than 20. A more positively-framed Discussion might leave readers with a more balanced appreciation for using RDS in their own studies of wheelchair users or other new populations.*

 Since our employment of RDS, a number of points, that may have improved our experience, have been considered. These include selecting more diverse seeds, and meeting with seeds to provide more information to them. To summarise these points, new text has been included our revised manuscript (see second paragraph in Discussion section).

# Peer review: Referee response for version 1

**DOI:** 10.5256/f1000research.9259.r14016

**Published:** 2016-06-13

**Authors:** John F Smith

**Affiliations:** 1 Postgraduate Tropical Medicine Program, Faculty of Medicine, Kohn Kaen University, Khon Kaen, Thailand

I support indexing this article as it as it appears to be a first-time test of the utility of Respondent Driven Sampling (RDS), a relatively novel sampling procedure, on a new "hidden" (hard to easily access) population--wheelchair riders.  In this case it was not successful in recruiting sufficient respondents, but reporting that, and possible reasons for that outcome, could be useful for refining usage of the procedure. 

 I think the title and abstract are clear and provide good insight into the major points of the paper.

 In addition to the possible reasons for low returns provided by the author I think it would be useful to include consideration of;

1. The typically low returns from internet surveys compared to face to face and pencil and paper surveys (see 1,2,3).  It is possible that promoting an internet survey was just insufficient to motivate the "seeds", or the second wave recruits, to recruit more members into the network, even with an iPad reward incentive.
2. Elaborating on the "stigmatization" issue raised in the discussion. The "mechanism by which perceived stigma may effect RDS sampling" rather than being unknown, may well be that other groups e.g., drug users, "men who have sex with men"(MSM), sex-workers etc as a result of stigma, may have stronger social networks and obvious gathering spaces and be easier to access via seeds.  Seeds with these groups are often "leaders", advocates who are "out", support group leaders, health service liaison/volunteer workers who move easily within those communities, social gatherings, entertainment or work spaces.  There are not the same social presses for wheelchair riders to have these group social connections or common activities.
3. Methodological point.  The article focuses on RDS's value for accessing representative population samples for research seeking normative and epidemiological data and concomitant importance of avoiding/controlling sample selection bias.  However, RDS could also be valuable for accessing qualitative data e.g., wheelchair riders' views/experiences with public policies/services etc where as wide a range of responses (variability) should be expected, indeed welcomed, rather than seen as requiring some statistical weighting or control procedure.  Other forms of criterion for data "completeness" e.g., saturation could be used here.  Another use for RDS is just to collect as large a sample as possible in hidden groups regardless of their representativeness.  For example, RDS "seeds" have been used to recruit as many MSM as possible in "test and treat" outreach campaigns for blood testing for HIV and ante-retroviral treatment programs.

               

 Overall a potentially useful addition to methodological literature on sampling procedures.

I have read this submission. I believe that I have an appropriate level of expertise to confirm that it is of an acceptable scientific standard, however I have significant reservations, as outlined above.

# Response

**Published:** 2016-08-23

**Authors:** Philip Schluter

**Affiliations:** University of Canterbury

Thank you greatly for your prompt, encouraging, and constructive feedback on our paper. Together with the comments made by the other two reviewers, your feedback has resulted in what we feel is a much improved paper.

 We would like to take this opportunity to make two notes. [1] The original submission was restricted to 1000 words in total – but, with permission from the journal, this constraint has been relaxed and we are able to include more material. This means we are able to broaden discussion of relevant issues and thus strengthen the manuscript. [2] This paper was submitted to the journal as an ‘observation article’, defined as: *Observation Articles allow the description of a novel observation that may be unexpected, and possibly currently without explanation. An observation can be a phenomenon that has been identified in field work, in the laboratory or through experimental analysis* (see: http://f1000research.com/for-authors/article-guidelines/observation-articles).

*I support indexing this article as it as it appears to be a first-time test of the utility of Respondent Driven Sampling (RDS), a relatively novel sampling procedure, on a new "hidden" (hard to easily access) population - wheelchair riders. In this case it was not successful in recruiting sufficient respondents, but reporting that, and possible reasons for that outcome, could be useful for refining usage of the procedure*.

 Thank you for recognising the novelty of this approach, together with your encouraging and supportive comments regarding our paper.

*I think the title and abstract are clear and provide good insight into the major points of the paper*.

 Thank you.

*In addition to the possible reasons for low returns provided by the author I think it would be useful to include consideration of*;

  

1. *The typically low returns from internet surveys compared to face to face and pencil and paper surveys (see 1,2,3). It is possible that promoting an internet survey was just insufficient to motivate the "seeds", or the second wave recruits, to recruit more members into the network, even with an iPad reward incentive*.

 

 Yes, this is indeed a valid consideration. In response, we have included a sentence the second paragraph in our revised discussion specifically referring to how more informed and enthusiastic seeds could have encouraged greater response rates, acting as a potential mitigation strategy against the impersonal nature of electronic surveys.

  

1. *Elaborating on the "stigmatization" issue raised in the discussion. The "mechanism by which perceived stigma may effect RDS sampling" rather than being unknown, may well be that other groups e.g., drug users, "men who have sex with men"(MSM), sex-workers etc as a result of stigma, may have stronger social networks and obvious gathering spaces and be easier to access via seeds. Seeds with these groups are often "leaders", advocates who are "out", support group leaders, health service liaison/volunteer workers who move easily within those communities, social gatherings, entertainment or work spaces. There are not the same social presses for wheelchair riders to have these group social connections or common activities.*

 

 Yes, this is valid and interesting point, and was also raised by another reviewer. In response, we have elaborated on the stigma issue by including new text in the first paragraph in the Discussion section addressing the area of perceived stigma, and whether or not wheelchair users have a sufficiently strong internal network that enable the random recruitment of other wheelchair users.

  

1. *Methodological point. The article focuses on RDS's value for accessing representative population samples for research seeking normative and epidemiological data and concomitant importance of avoiding/controlling sample selection bias. However, RDS could also be valuable for accessing qualitative data e.g., wheelchair riders' views/experiences with public policies/services etc where as wide a range of responses (variability) should be expected, indeed welcomed, rather than seen as requiring some statistical weighting or control procedure. Other forms of criterion for data "completeness" e.g., saturation could be used here. Another use for RDS is just to collect as large a sample as possible in hidden groups regardless of their representativeness. For example, RDS "seeds" have been used to recruit as many MSM as possible in "test and treat" outreach campaigns for blood testing for HIV and ante-retroviral treatment programs.*

 

 The value of using the RDS in qualitative research is a very interesting concept, and could be raised in the Discussion. However, on balance we feel it may be beyond the scope of our paper here, which was to discuss our experience of unsuccessfully satisfying the requirements needed to produce a theoretically representative sample of the population with no sampling frame.

*Overall a potentially useful addition to methodological literature on sampling procedures.*

 We appreciate these words and the additional references offered.

# Peer review: Referee response for version 1

**DOI:** 10.5256/f1000research.9259.r13572

**Published:** 2016-05-09

**Authors:** Jesse Kokaua

**Affiliations:** 1 Pacific Islands Research & Student Support Unit, University of Otago, Dunein, New Zealand

I think this paper has merit as a scientific publication, in that it adds to the body of research about RDS by its application to a "hidden" population, that by all accounts should be reasonable, but turned out to be not effective.

The abstract begins with a sentence that ends with a repeated point "rapid increasing life-span" and "accelerated general population ageing". One of those statements is redundant. Paragraph 2 of the introduction starts with international findings but does the second sentence beginning with "Despite this . . " refer to the NZ experience, or outside of the US and UK?

I feel that some points could be expanded upon:

The authors could expand, in the introduction, how the method improves the validity of, and reduces biases in, population-based estimates over other methods.

Examples of typical target populations for this method that are introduced in the discussion should move to the introduction, as it supports the novelty of this application.

I feel the results are two-fold. Firstly, the sample as described. I know its a moot point but I would be interested to know how big a sample is required to reach equilibrium.

 Secondly, since this is a paper on the RDS, I would have liked to see a description of the total numbers of people approached in each wave, with eligible and ineligible participants. Thus reporting if the failure was a result of response, eligibility, or simply a lack of invitation to participate in subsequent waves.

This is an interesting application of a novel method of sampling from a group of people made hard-to-reach, it seems, as a result of their successful integration into contemporary New Zealand society. One characteristic of strongly stigmatised groups in New Zealand, such as those identified in the paper, are their tendency to establish strong internal networks, both informal and formal, that I feel would be useful to this type of methodology.  It would be difficult for individuals with small networks to fulfill the random selection criteria for RDS.  I take it that this may have influenced the less stigmatised wheelchair user population but it wasn't clearly stated if this was the case.

The paper reports a good example of where a useful method may not apply, in its initial format, to a particular interest group.However, it wasn't totally discounted as a potential approach but the reader is left without any suggestion as to how it might be modified to improve its take up, except for the suggestion of formative research, while fulfilling the assumptions required for RDS to provide unbiased population-wide estimates.

I have read this submission. I believe that I have an appropriate level of expertise to confirm that it is of an acceptable scientific standard, however I have significant reservations, as outlined above.

# Response

**Published:** 2016-08-23

**Authors:** Philip Schluter

**Affiliations:** University of Canterbury

Thank you greatly for your prompt, encouraging, and constructive feedback on our paper. Together with the comments made by the other two reviewers, your feedback has resulted in what we feel is a much improved paper.

 We would like to take this opportunity to make two notes. [1] The original submission was restricted to 1000 words in total – but, with permission from the journal, this constraint has been relaxed and we are able to include more material. This means we are able to broaden discussion of relevant issues and thus strengthen the manuscript. [2] This paper was submitted to the journal as an ‘observation article’, defined as: *Observation Articles allow the description of a novel observation that may be unexpected, and possibly currently without explanation. An observation can be a phenomenon that has been identified in field work, in the laboratory or through experimental analysis* (see: http://f1000research.com/for-authors/article-guidelines/observation-articles).

*I think this paper has merit as a scientific publication, in that it adds to the body of research about RDS by its application to a "hidden" population that by all accounts should be reasonable, but turned out to be not effective*.

 Thank you for your positive comments and summation. This, for us, was the basis for opting for the “novel observation” article type.

*The abstract begins with a sentence that ends with a repeated point "rapid increasing life-span" and "accelerated general population ageing".* *One of those statements is redundant.*

 Although the intention was to make two important points, the first referring to wheelchair users and the second referring to the general population, we agree that this is unnecessarily confusing. We have revised this sentence to: “Internationally, wheelchair users are an emerging demographic phenomenon, due to their rapidly increasing life-span.”

*Paragraph 2 of the introduction starts with international findings but does the second sentence beginning with "Despite this . . " refer to the NZ experience, or outside of the US and UK?*

 This appears to be an international trend. However, to be clearer we have included “in New Zealand and Australia” and inserted an apposite reference.

*I feel that some points could be expanded upon: The authors could expand, in the introduction, how the method improves the validity of, and reduces biases in, population-based estimates over other methods.*

 Agreed – indeed, a similar point was raised by another reviewer. Our original submission was limited in scope due to the word restrictions, as outlined above. However, in this revision, we have included more information and discussion. Specifically, we have included text providing a brief introduction to RDS (See second paragraph in Introduction section).

*Examples of typical target populations for this method that are introduced in the discussion should move to the introduction, as it supports the novelty of this application.*

 Thank you for this comment; it indeed strengthens our introduction. In our revised manuscript we have included the following sentence with paragraph one of the Introduction: “RDS has traditionally been used to sample ‘hidden’ populations with inadequate sampling frames, such as those with greater risk of HIV, including injecting drug users.”

*I feel the results are two-fold. Firstly, the sample as described. I know it’s a moot point but I would be interested to know how big a sample is required to reach equilibrium.*

 We also felt that this information would strengthen our paper, but omitted it initially – due to the word count restrictions. In this version, within the Introduction, we now explicitly add an explanation about the concept of equilibrium and how it is reached (see third paragraph), while the second paragraph in the Results section explains specifically why our sample failed to satisfy the threshold for in-equilibrium data.

*Secondly, since this is a paper on the RDS, I would have liked to see a description of the total numbers of people approached in each wave, with eligible and ineligible participants. Thus reporting if the failure was a result of response, eligibility, or simply a lack of invitation to participate in subsequent waves.*

 These details would be hard, if not ethically impossible, to determine. Consistent with RDS protocol, an email was sent to people asking them to send on the invitation to three people, but there was no obligation for us (the researchers) to receive confirmation about whether all three recruitment codes were successfully allocated and distributed. Nevertheless, more information has been included in the Methods section (second paragraph) regarding how participants were asked to recruit three others, and the first paragraph in the Results section, which clarifies recruitment and response rates.

*This is an interesting application of a novel method of sampling from a group of people made hard-to-reach, it seems, as a result of their successful integration into contemporary New Zealand society. One characteristic of strongly stigmatised groups in New Zealand, such as those identified in the paper, are their tendency to establish strong internal networks, both informal and formal, that I feel would be useful to this type of methodology. It would be difficult for individuals with small networks to fulfil the random selection criteria for RDS. I take it that this may have influenced the less stigmatised wheelchair user population but it wasn't clearly stated if this was the case.*

 Yes, this is valid and interesting point and was also raised by another reviewer. In response, we have included two sentences in the first paragraph of the Discussion section addressing the area of perceived stigma and whether or not wheelchair users have sufficiently strong internal networks that enable the random recruitment of other wheelchair users.

*The paper reports a good example of where a useful method may not apply, in its initial format, to a particular interest group. However, it wasn't totally discounted as a potential approach but the reader is left without any suggestion as to how it might be modified to improve its take up, except for the suggestion of formative research, while fulfilling the assumptions required for RDS to provide unbiased population-wide estimates.*

 Thank you for this comment that will strengthen our paper. In response we have included more information in the second paragraph of the Discussion section about potential ways to improve similar studies, namely, selecting diverse seeds with larger networks and providing greater information and education to those seeds.
